# Cancer neuroscience and glioma: clinical implications

**DOI:** 10.1007/s00701-024-06406-2

**Published:** 2025-01-03

**Authors:** Manfred Westphal, Richard Drexler, Cecile Maire, Franz Ricklefs, Katrin Lamszus

**Affiliations:** 1 Institute for Tumorbiology, University Hospital Hamburg Eppendorf, W29 - R34, Hamburg, 20246 Germany; 2 Department of Neurology and Neurological Sciences, Stanford University, Stanford, CA USA; 3 Department of Neurosurgery, University Hospital Eppendorf, Hamburg, Germany

**Keywords:** Glioblastoma, Methylation profiles, Neural signature, Immunosuppression, Extracellular vesicles, Neuroglial communication

## Abstract

In recent years, it has been increasingly recognized that tumor growth relies not only on support from the surrounding microenvironment but also on the tumors capacity to adapt to – and actively manipulate – its niche. While targeting angiogenesis and modulating the local immune environment have been explored as therapeutic approaches, these strategies have yet to yield effective treatments for brain tumors and remain under refinement. More recently, the nervous system itself has been explored as a critical environmental support for cancer, with extensive neuro-tumoral interactions observed both intracranially and in extracranial sites containing neural components. In the brain, interactions between glioma cells as well as metastatic lesions with neural components have clinical implications for diagnostics, risk assessments, neurological sequelae, and the development of innovative therapeutics. Here, we review these neuro-tumoral dynamics, emphasizing aspects relevant to neurosurgical practice.

## Introduction

Our understanding of glioblastoma biology, and glioma in general, is undergoing a transformative shift. Once termed “glioblastoma multiforme” to reflect its complex and heterogeneous appearance to neuropathologists, neuroradiologists and neurosurgeons for decades, the term has since been streamlined, molecularly defined and shortened to “glioblastoma” [41]. Rather than succumbing to the overwhelming “multiforme” nature of glioblastoma, previously deemed too complex for a unified therapeutic approach, modern analytical methods and biomathematical dissection have unraveled increasingly granular layers of this complexity, revealing underlying organizational principles. Key concepts such as phenotypic synchronicity [44, 71], adaptability [49] and multilayered cellular organization [23] along neuro-developmental patterns [5, 6] have emerged, providing a more structured understanding of the disease.

In parallel, the bidirectional interactions between tumor and the host microenvironment have become a central focus. Whereas earlier research predominantly targeted cellular oncobiology to uncover therapeutic opportunities, the ability of the tumor to thrive from interaction with the host environment, adopting a neurocentric view [12] and a neurobiology of glioma [87] has highlighted the critical role of the glio-neuronal crosstalk, a phenomenon that can be seen as a “neural adaptive mimicry” (NAM) of tumor cells. This environmental adaptation grants glioma cells a survival advantage [45, 79]. Notably, neuro-adaptive mechanisms are not exclusive to intrinsic tumors; cerebral metastases from different cancers also leverage neural interactions for seeding and survival [57, 92, 96, 99, 102].

Unfortunately, but unavoidably, the increments of conceptual understanding of simultaneously present cellular phenotypes and adaptive dynamics outpaces the translational capacity of neuro-oncology and neurosurgery for therapeutic innovation. Nonetheless, these new insights already have some present or potentially fast emerging clinical implications in the field of neuro-oncology. In this context, there are some implications specifically also for neurosurgery, like risk adaptation or tailored anticonvulsive treatment [16], which will be further detailed herein.

### Prognosis and risk assessment with molecular signatures

In respect to defining predictors of survival, brain tumor neuropathology – particularly for glioblastoma - underwent a transformative shift when epigenetics were included in the tumor assessment and classification [52]. The distinction between methylated and unmethylated MGMT promoter status has long been established as a clinical practice [27]. Extending epigenetic methylation analysis across the whole genome led to the development of a CNS tumor methylation classifier which is currently in global use [10] and is a fixed element of discussion in interdisciplinary tumor boards. This advancement has allowed for more precise allocation of tumors to molecularly defined groups, independent of their morphological appearance which allows for more precise entry criteria into current and future clinical trials and also potentially enables retrospective re-evaluation of past trial results for predictive markers [11, 64]. For example, conclusions from clinical trials including a significant proportion of “oligo-astrocytoma” must now be reconsidered, because of the elimination of this entity by molecular genetics [63] and its non-existence in the methylation classifier. Until its elimination it has undoubtedly embraced diverse entities, now seen to have vastly different prognoses.

Importantly, the CNS methylation classifier has further enabled bioinformatic dissection of glioblastoma into subtypes defined by methylation signatures, such as the receptor tyrosine kinase (RTK) types I and II and the mesenchymal subtype (MES) [10]. The mesenchymal signature, in particular, is thought to have the most substantial contribution from the microenvironment and from dynamics in response to therapies [8]. Clinical-correlation studies of the RTK I and RTK II glioblastoma subclasses have shown that the RTK II subgroup is associated with a higher risk of seizures [59]. More importantly, complete resection has a significant prognostic impact on overall survival [17] in RTK I and II subtypes but not in the MES subtype. Obviously, timely information on the molecular subclass would allow clinicians surgical risk stratification for cases where increasing extent of resection bears a risk of neurological harm, a risk not worth taking when the radicality is of diminished relevance. This current conclusion of the impact of extent of resection wasbased on correlation of methylation subclass to gross total resection (GTR) as obtained in the available data set. (GTR) will universally remain the primary surgical goal for some time, and serve as main correlative comparator for clinical trials. Albeit, supramarginal resection is currently an evolving concept [103](see below) so this analysis needs to be re-evaluated and validated in suitable cohorts with more extensive resections to see whether the effect of methylation subclass on extent of resection is maintaned. It can be safely assumed, that, had the classifyer been available in the past, correlative analyses of the RTK subtype with surgical outcomes in historical series would have provided a clearer answer regarding the prognostic value of extent of resection, potentially resolving longstanding conflicts in the literature on this topic [55, 65, 72, 75].

While the RTKI/RTKII/MES distinction has already shown clinical relevance, further refinement in methylation analyses allows to propose more comprehensive patterns and the concept of signatures. These signatures assess tumor relationships to known tissue-specific methylation profiles or identify the contributions of such signatures within a tumor mass. Methylation-based signatures can be either organotypic or cell type specific. For various tissue, signatures based on methylation patterns – even down to the single-cell level - have been established and compiled into universally accessible atlases [2, 39, 42, 51, 75].

**Fig. 1:**
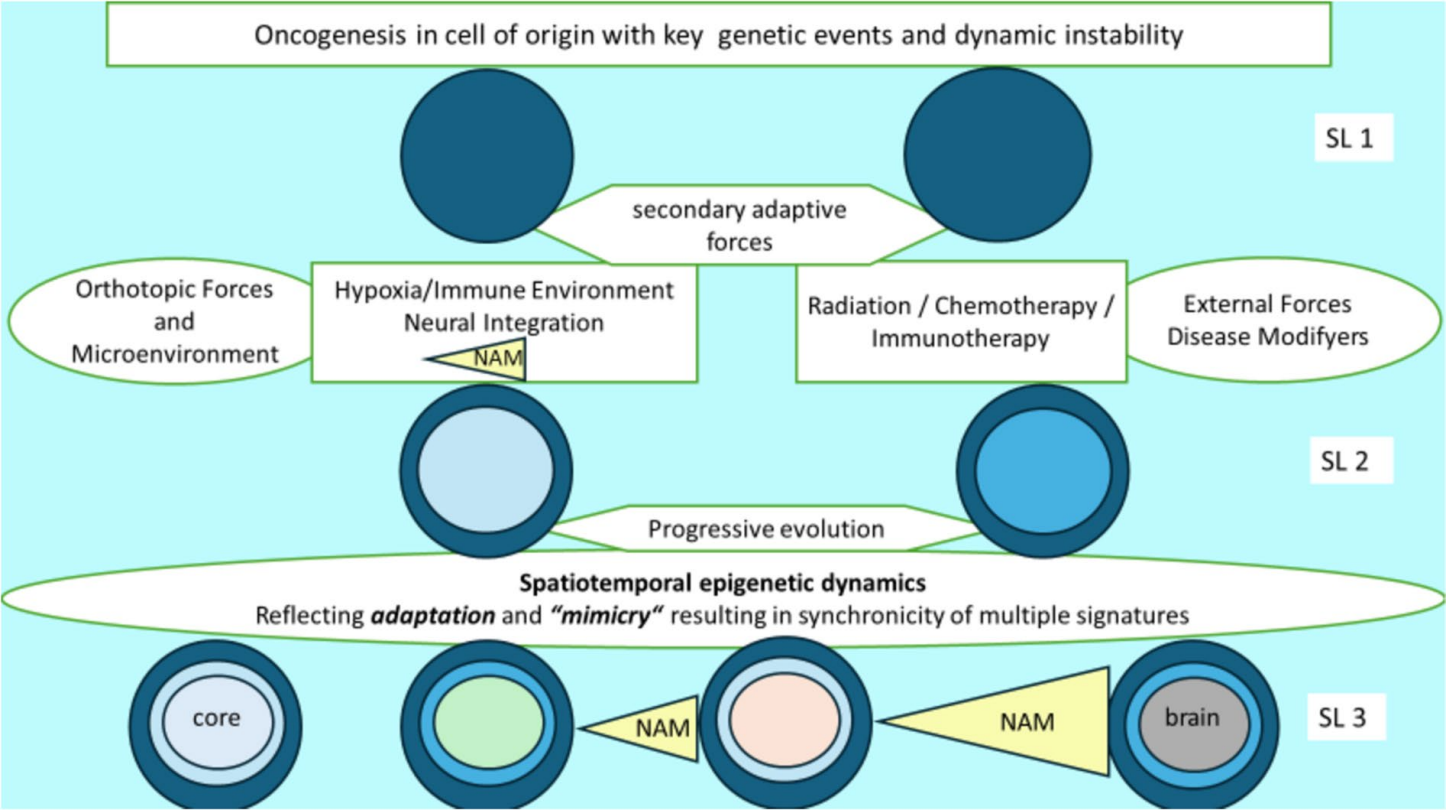
Conceptual description of the path to the synchronicity of multiple genomic /epigenetic signatures resulting in diverse cellular programs within GBM. Hierarchically, after the initial oncogenic event resulting in the initial signature level (SL 1), mostly genetics based, the further evolving gene-expression / epigenetic signatures will be modified by local selective pressure such as hypoxia or external pressures like radiation and chemotherapy (SL 2). In the definitive, multilayered glioblastoma with increasing radial distance from the necrotic core, infiltration into the brain and fending off the immune surveillance result in increasingly complex adaptive signatures also by acquisition of neural characteristics (neural adaptive mimicry, NAM) with increasing tumor vitality and subsequent poor patient survival. (Adopted in principle from Greenwald et al. [23], referring to gene expression signatures.)

This approach allowed to propose a “neural” signature and to recently interrogate the methylation profile of gliomas from multiple consortial databases [19]. Using this neural-like signature and defining it arbitrarily as “high” or “low” within a dataset of over 5,000 patients, it was found that a high neural-like signature was significantly associated with poorer overall survival [19]. The predictive power of “neural features” as such was already recognized earlier by clinical correlations of ion channels expression [56, 70, 77, 84]. In a biological context, this is to be interpreted as an advantageous adaptation to exploit microenvironmental interactions resulting in the previously mentioned NAM which has been firmly established to generate survival advantages for glioma cells [37, 53, 73, 78, 79, 81]. Conceptually this would ideally call for the additional assessment of epigenetic profiles to the neuropathological evaluation of glioblastoma. The dynamic epigenetic plasticity among different glioma cell populations [18] is to be seen as superimposed on the oncogenetic programs of gene mutations, amplifications, fusions or deletions [49] and a highly granular map exposing all therapeutic vulnerabilities might then emerge (Fig. 1). Looking ahead, the assessment of NAM as an additional prognostic parameter may complement the highly insightful and widely used distinction of four glioblastoma states into oligodendrocyte-precursor like (OPC), neural-precursor like (NPC), mesenchymal like (MES) and astrocyte-like (AC) which was generated by integrated single cell RNA sequencing and genetic and expression analyses and highlights the plasticity between these states [49]. We face a growing complexity of analytical layers originally resulting in the distinction of pro-neural, neural, classical, mesenchymal subtypes by gene expression analysis [82], later dropping the “neural” subtype [85] with its further refinement by methylation profiling [52]. As complex and detached from surgical reality as it appears, the currently emerging build-up of layers of complexity reflects the necessary level of understanding required to improve treatment of glioblastoma in daily clinical practice.

The generation of methylation signatures from complex tissues through extraction and subsequent deconvolution requires advanced technological infrastructure, which will take time to implement in routine clinical practice. At present, the highly promising result of the predictive power of the neural signature was generated from large international databases, extracting its information from tissues which were collected in a non-standardized way, rather than through a single, stringent protocol (see below). Consequently, the degree of contributions of the many different cell components within each specimen of the cohorts remains unknown. Tumor cells, vascular cells, immune cells, stromal cells with a vast array of cellular signatures contribute to the overall datasets. Nevertheless, bioinformatic techniques, such as deconvolution, have been developed to estimate the relative contribution of a “neural” signature [1, 14, 46]. Several proposals for deconvolution of glioma datasets have been proposed, and it is expected that this process will undergo refinement, validation and eventually standardization as the essential components of these signatures are delineated. This bioinformatic process introduces a new dimension to traditional pathology and immunohistochemistry for markers like the predominant IDH-1 mutations, ATRX loss, assessment of defined molecular features like EGF-R amplification, or copy number variations, serving as the foundational “baseline signature” [86] (Fig. 1). Likely, molecular data and signatures will increasingly be used to train artificial intelligence on images, both radiological and pathological, so that with some accuracy, important information may be obtained preoperatively or rapidly thereafter [36]. In the case of IDH mutations in astrocytoma and oligodendroglioma, proof of concept in radiomics has already been demonstrated [95], but will be more challenging for glioblastoma, which by definition is IDH wildtype.

Neural adaptive characteristics have also been identified across a broad histological spectrum of brain metastases [66, 93] specifically from breast [50, 96] and melanoma [9], with important implications for the current neurosurgical strategies [34]. It is well established that en bloc resection in toto, when possible, is preferable to a piecemeal resection - not only for achieving complete resection but also minimizing the risk of meningeal seeding [74]. With the concept of generation of a favourable immediate microenvironment at the interface between metastases and brain, removal of a small rim of adjacent edematous brain may be warranted to minimize local recurrence. To better understand the consistency of neural adaptation across different histological entities and in patients with multiple metastases, that peritumoral zone should be investigated whereever feasible, providing insights into tumor-induced microenvironmental changes and a subsequent pro-tumorigenic or tumor supportive milieu. In most cases, as shown in studies on surgical methodology, this approach is considered safe [54].

### Biological significance of signatures

Cellular signatures primarily reflect tumors´ opportunistic adaptions to the microenvironment, demonstrating their cellular potential to interact and manipulate the surroundings. Through NAM, glioma cells can integrate into neural circuits, exploiting supportive neural signals [79, 94]. These interactions are both neurochemical [80] and mediated by soluble factors and opportunistic receptor expression, such as GDNF or Neuroligin 3 [81]. Conceptionally, disrupting these tumor-specific crosstalk pathways offers new therapeutic opportunities [101].

Emerging therapeutic approaches include pharmacological interferences that exploit glutamatergic signaling pathways by antiepileptics drugs (AED). Even before the comprehensive description of the neuro-glioma synaptic integration, glutamatergic signaling was in the focus of early clinical trials, including the AMPA receptor antagonist talampanel [24]. Presently another more advanced anti-glutamatergic agent, perampanel, is in clinical trials [29]. With the growing focus on NAM, integrating signatures of the respective tumor tissue into the correlative data analysis may show the functional relevance of glutamatergic signaling for overall survival.

The awareness of neural adaptation in glioblastoma has also influenced drug screening strategies. In a recent high-throughput screening of over a million compounds, a „neuro-active“ profile was prioritized [98], highlighting the option of repurposing AEDs for glioma treatment. AEDs are used to mitigate glutaminergic hyperexcitability, which drives glioma cell proliferation. In contrast, AEDs working via enhancing GABAergic activity will be of no use and even contraindicated as GABAergic input is also supporting tumor growth [22]. The identification of novel anti-epileptic therapeutic targets has unveiled numerous therapeutic vulnerabilities within glioma, particularly involving cholinergic, adrenergic, dopaminergic, and serotonergic receptors [104]. More transmitter interfering approaches are to be expected with the caveat, that interfering selectively with these systems is challenging because of the integration of glioma innervating neurons (GIN) into regional or even distant neural networks [97]. However, evidence suggests that the electrophysiological characteristics of GINs may be different from non-glioma innervating neurons from the same areas of origin [97] and it remains to be explored whether that can be translated into therapeutic opportunities. Importantly, therapies targeting the opportunistic exploitation of synaptic transmission by glioma cells, should be viewed as adjuncts to existing base-line oncogenic programs [44]. Given the neural signature superimposed upon the baseline oncogenic program, chemotherapy will likely remain a foundational treatment, while potential drug interactions are still under investigation.

Additionally, cells with a neural signature appear to significantly modulate the immune environment, which is highly relevant to the numerous immunotherapeutic efforts in glioblastoma. The contribution of neural-like signaling to an immunosuppressive tumor environment correlates with poor survival across cancers [13]. In glioblastoma, the presence of neuronal input facilitates functional connectivity between glioblastoma cells and the host brain networks resulting in an immunosuppressive environment [100], partially resulting from neuronally well connected tumor cells producing synaptogenic thrombospindin-1. Nonetheless, the specific signaling cascades involved in the cross-talk between glioblastoma cells, glutamatergic neurons, and immune cells remain to be elucidated [100].

### Neurosurgical implications

Further understanding of the neuroadaptive mimicry in glioblastoma will necessitate changes in therapeutic approaches because of the simultaneous presence of different phenotypes. This, representing the main reason for therapeutic frustration, commonly referred to as „heterogeneity“ is tumor-biologically to be seen as a synchronicity of different transcriptional programs across different tumor regions and special adaptations in the infiltration zone [26, 44]. This comes intrinsically with regional and likely also individually inconsistent susceptibilities to therapies, - unless they address the underlying oncogenic events, - what so far has proven to be ineffective and frustrating [4].

A key neurosurgical task in the current retrenching to map the synchronously present signatures is selective and well annotated sampling. Several examples exist for the power of such approaches [44, 71]. Recently, a proposal has been made for comprehensive tissue sampling by an international consensus [35]. At present, this may be considered a step back, but the present evolution of signatures, specifically the NAM, needs to be validated for consistency and correlation with clinical parameters such as anatomical location and zones of infiltration and imaging characteristics. Quality of tissue sampling will have to be factored into correlative tissue analysis of clinical trials with drugs interfering with neural signaling as that neural program is likely spatially restricted and transient to allow for invasion and colonization of the brain by the cells of the penumbra, so core tissue may give false negative correlations because samples taken out of the center may not predict the true drug target availability in the infiltrative zone. Likewise, longitudinal sampling throughout recurrence and probably even multiple recurrences has to go with the same comprehensive sampling to be able to show possible therapy-induced adaptive dynamics of signatures [18]. Centers which are enabled to perform “window of opportunity trials“ may contribute to the issue of drug distribution if a drug is administered preoperatively and distribution assessed shortly after and correlated not only to perfusion characteristics but also the signatures from targeted sampling across the diverse tumoral and peritumoral zones.

One prerequisite for the comprehensive regional profiling is the provision of tissue also from the FLAIR region. In this context, the concept of supramarginal resection appears perfectly suited for obtaining information on the critical invasive cells outside contrast enhancement. While supramarginal resection has been refined rather for low-grade gliomas with consideration of connectomes and complex functional testing in awake surgery [20], it gets extrapolated to glioblastoma and even lobectomies when feasible are considered again as recently reviewed [3]. Naturally the limitation in the reviewed series is to right frontal and temporal locations [61, 67] but it indirectly confirms the crucial relevance of the penumbra cell population. Accordingly, also the degree of invasiveness has recently been correlated to increase in survival when supramarginal resections are performed [76]. On the one hand that proof is reassuring in justifying to performe aggressive surgery, but on the other hand it must be considered that in contrast to diffuse low-grade tumors, with glioblastoma there is less time for plasticity-based “rewiring” of the brain. Thus, supramarginal strategies will be limited where speech, memory and motor functions are not inside the immediate infiltration zone starting at the edge of contrast enhancement and can be monitored. Also, some neuropsychological qualities like emotional reactivity will be difficult to test even in the awake situation [33], so there are also voices seeing that supramarginal concept critical [25] illustrating the highly diverse views on “eloquence”.

Another prerequisite in the context of neural signature assessment in the tumoral penumbra is the refinement of analytical technology as the density of tumor cells in the healthy appearing infiltration zone is low, calling for single cell analyses which will be challenging to become routine for some time.

Apparently, from current studies with spatial resolution it appears as if the infiltrative edge is the zone in which the tumor afforded mimicry is translated into a host-interactive advantage. Local therapies have mostly aimed to take oncotherapy to the borders, to reach some additional therapeutic effect adding to resection using BCNU-wafers [89], adenoviral prodrug converting gene therapy [90], oncolytic virus [48] or intracavitary radioimmunotherapy [62]. From the current thinking it is conceivable that interfering with neuro-adaptive mechanisms at the infiltrative edge may be an additional new targeted perspective for local therapy once we know the mechanisms and have stable substances penetrating far enough away from the edge.

In essence, the opportunity for surgical risk adaptation according to RTK/MES status and the clinical prognostic relevance of tissue from appropriately collected specimens and future drug selection according to deconvoluted signatures call for neurosurgical integration of these current developments. An ideal situation in which a tailored aggressiveness can be ascribed to each patient, will be linked to refinement of techniques to preoperatively assess predictors, which at least could reflect the RTK type, and the extent of NAM as assessed by the neural methylation signature. This leads to the efforts of biomarker development which will be briefly touched.

### Signatures and biomarker development

The liquid biopsy field has entered also neuro-oncology with many potential applications [69, 83, 88]. Cell free DNA (cfDNA) from serum has become a workhorse in general oncology to look for specific mutations [7]. In addition, circulating tumor cells are a very useful tool in clinical oncology, but for glioblastoma of limited applicability [47]. Paradigmatic mutations found in cfDNA for glioblastoma like the EGF-RvIII or TERT mutations or copy number variations would be of bona fide value as biomarkers with the caviat of yet unsatisfactory detectability.However, they only indicate the baseline of transformative oncogenic events onto which the epigenetic signatures and subsequent expression signatures are superimposed. Extracellular vesicles appear attractive as a source of information as intra- and extravesicular cargo seems to be able to reflect the cellular methylation pattern, at least from cells in culture [43]. The analysis of EVs from serum is still burdened with the quest for adequate selective purification of tumor derived EVs with distinctive markers. In addition, it has not yet been proven, that the origin of the DNA attached to GBM-EVs is definitively and exclusively from the living or dying cells of presumed origin. The numbers of EVs in serum are clearly associated with tumor burden, and extent of FLAIR [60], but likely reflect the origin from both, all cells in the tumor as well as its reactive surroundings [91]. Thus, future work has to correlate representative tissue sampling, harvesting of tissue EVs and correlation of findings with serum and when possible CSF EVs. How granular analysis of EVs and their reflection of signatures can get will have limits, as specific signatures like in hypoxic „spots“ [23] will enter the bulk of EVs and it will be a deconvolutional task to push the limits of signature distinction. With refinement of isolation and single EV techniques in progress, it will still take some time before EVs enter a biomarker routine. With the EVs reaching the blood stream via a postulated glymphatic system in the brain and connectivity of the glymphatics with the CSF, the analysis of CSF to look for marker molecules, specifically cfDNA [32] for tumor is another way to try and capture as much information on a tumor signature as possible [68].

In parallel to efforts to preoperative marker assessment, promising intraoperative sequencing technologies are in the state of validation such as nanopore sequencing [15] which in combination with computational techniques shows promise to provide real time guidance [28]. Raman spectroscopy has already been refined to very accurately define the cellular composition at the border of resection [21]. It has to be seen whether integration and training of artificial intelligence will at some point pick up the critical cellular signatures when spectra are adequately deconvoluted [30, 31, 38, 40, 58].

In conclusion, the emerging insights into the factual neurobiology of glioblastoma have led to a deeper understanding of biological roots for tumor evolution and behavior. New neural centered pharmacological therapeutic opportunities have arisen as an additive to current therapy of the known oncotargets. In addition, the degree of NAM, captured by a methylation signature with evolving pre- and intraoperative technologies will allow for surgical risk adaptation based on epigenetic profiling resulting in optimal safe resections. In essence, integrating the new conceptual understanding of tumor-host interactions should lead towards a neuroscience informed surgery of brain tumors.

## Data Availability

No datasets were generated or analysed during the current study.
